# The Story of the Insane from Year to Year

**Published:** 1896-08-15

**Authors:** 


### THE STORY OF THE INSANE FROM YEAR TO YEAR.

### Ninth Series.

### THE CORK DISTRICT ASYLUM.

As usual in the reports of Irish asylums, we find the limits of accommodation given, a course which we have ere now recommended English asylums to adopt. Here there is room for 1,200 patients; but on the first day of the year the numbers were 221 in excess of this; and at the close of the year there were 1,259 patients in residence. There were 284 admissions, 107 recoveries, 25 discharged relieved, and 107 deaths. Calculating the percentages in the way usual in asylums, the recoveries stand at 37·6 and the deaths 8·5. Of the deaths 22 were caused by brain diseases, and the very large number of 39 died of consumption, and we think this high mortality should be investigated, accounted for, and, if possible, prevented. Dr. Woods seems to blame the heating arrangements; but we doubt if this will account for it. Turning to the table showing the causes of insanity in the year's admissions, we find drink heading the list with 47; previous attacks account for 42, and hereditary tendency for 30. When referring to the admissions, Dr. Woods says that the Irish whisky is adulterated with a highly exciting foreign spirit which has an injurious effect. This is very likely, and the remedy lies in abstaining from the whisky altogether. As regards hereditary predisposition, we are pleased to quote Dr. Woods' opinion, and we hope it may sink deep into the hearts of his own countrymen and ours too: "I cannot avoid," he says, "once again referring to the number of patients admitted with hereditary predisposition. If the lower classes would only take more trouble in selecting their wives we would have much less insanity in the country; few can realise the number of intermarriages there are in some districts. Men who have members of their families insane should, under no circumstances, intermarry with relatives. It is not sufficiently known how nearly allied are the various neurotic diseases one to another, and how a father suffering from one form will, in all probability, transmit a worse disease to his offspring, unless he marries into a perfectly healthy family. Much has been written during the last year with regard to assaults committed by patients who have been too soon discharged from asylums—far greater injury is, I believe, done by these patients begetting children very liable to be epileptic, imbecile, insane, drunkards, or criminals." We are not so satisfied with what Dr. Woods says about the advisableness of having detached hospitals for the treatment of acute cases. It has never yet been shown that more cases would be cured thereby, and if it be true it is not the way to reduce insanity in future generations, for, although a medical man is bound to do all he can to cure his cases, it is only too certain that every cure in the present will increase the insanity of the future. We believe the Germans gave the hospital system a trial, and are now abandoning it. We learn that the committee appointed a man as head attendant who had no previous experience in asylums, and the inspector well says the board have in so doing assumed a grave responsibility. The head attendants and all the subordinate staff should be appointed by the medical superintendent, and until this is the rule there will never be perfect order in the institution. The annual cost is £19 per head per annum.

